# Epigenetic reprogramming in metabolic dysfunction–associated steatotic liver disease: from metabolic memory to precision medicine

**DOI:** 10.3389/fphys.2026.1791638

**Published:** 2026-03-19

**Authors:** Basile Njei, Yazan A. Al-Ajlouni

**Affiliations:** 1 Engelhardt School of Global Health and Bioethics, Euclid University, Bangui, Central African Republic; 2 Section of Digestive Diseases, Department of Medicine, Yale University, New Haven, CT, United States; 3 Artificial Intelligence Programme, University of Cumbria, Carlisle, United Kingdom; 4 Ohio University Heritage College of Osteopathic Medicine, Athens, OH, United States; 5 Yale Liver Center, Yale New Haven Health, New Haven, CT, United States; 6 Yale International Medicine Program, Yale University, New Haven, CT, United States; 7 Department of Rehabilitation, Montefiore Medical Center/Einstein School of Medicine, Bronx, NY, United States

**Keywords:** DNA methylation, epigenetics, metabolic dysfunction associated steatotic liver disease, microRNA, precision medicine

## Abstract

**Background:**

Metabolic dysfunction associated steatotic liver disease (MASLD) is the most common chronic liver disease worldwide and is characterized by substantial heterogeneity in clinical presentation, disease progression, and treatment response. Conventional metabolic risk factors do not fully explain this variability. Epigenetic regulation has emerged as a central mechanism linking metabolic stress to sustained alterations in hepatic gene expression and long-term disease behavior.

**Methods:**

This narrative review synthesizes evidence from human observational studies, interventional studies, systematic reviews, and experimental research examining epigenetic regulation in MASLD. Key epigenetic mechanisms reviewed include DNA methylation, histone modifications, and noncoding RNA mediated regulation, with emphasis on physiological relevance and translational implications.

**Results:**

Epigenetic alterations in MASLD are closely associated with chronic metabolic stress and influence pathways involved in lipid metabolism, insulin resistance, inflammation, mitochondrial dysfunction, and fibrogenesis. These changes contribute to disease persistence, progression, and heterogeneity, including lean disease phenotypes. Evidence suggests partial reversibility of epigenetic programming following lifestyle modification, metabolic improvement, and surgical intervention. Circulating microRNAs and DNA methylation signatures show promise as noninvasive tools for disease phenotyping, risk stratification, and monitoring, although clinical validation remains limited.

**Conclusions:**

Epigenetic reprogramming represents a core biological process in MASLD that integrates metabolic exposures with long term hepatic outcomes. Improved understanding of epigenetic plasticity and stability across disease stages may inform earlier intervention strategies and support the development of precision medicine approaches in this heterogeneous condition.

## 1 Introduction

Metabolic dysfunction associated steatotic liver disease (MASLD) is the most common chronic liver disease worldwide and encompasses a wide spectrum of clinical and biological phenotypes (Corrao et al., 2025). MASLD is closely linked to obesity, insulin resistance, and type 2 diabetes mellitus and represents a growing global health burden affecting both adult and pediatric populations (Bril et al., 2024; Jiang et al., 2024; Mejía-Guzmán et al., 2025). Recent epidemiologic data indicate increasing prevalence among adolescents and young adults, underscoring the urgency of improved mechanistic understanding and early risk stratification strategies (Nairz et al., 2024; Mejía-Guzmán et al., 2025).

The pathogenesis of MASLD is complex and multifactorial and is commonly conceptualized through the multiple hit framework, which integrates genetic susceptibility, environmental exposures, dietary patterns, and metabolic dysfunction as interacting drivers of disease initiation and progression (Jiang et al., 2024; Zargar et al., 2025). Insulin resistance plays a central role in this process and contributes to altered lipid handling, hepatic inflammation, and fibrogenesis (Bril et al., 2024; Mejía-Guzmán et al., 2025). However, these factors alone do not fully explain the marked heterogeneity observed in clinical outcomes, disease severity, or response to therapeutic interventions.

Epigenetic regulation has emerged as a key biological mechanism linking metabolic stress to sustained alterations in hepatic gene expression. Epigenetic processes including DNA methylation, histone modifications, and non-coding RNA mediated regulation influence transcriptional programs without altering DNA sequence and are essential for maintaining liver metabolic homeostasis (Takahashi et al., 2023; Wang et al., 2024; Li et al., 2025). In MASLD, epigenetic dysregulation has been shown to affect pathways involved in lipid metabolism, inflammation, mitochondrial function, and cellular stress responses, thereby contributing to disease onset and progression (Caputo et al., 2023; Takahashi et al., 2023; Wang et al., 2025).

Several studies have highlighted the role of epigenetic regulation in hepatic lipid metabolism. Genes such as TCF7L2 have been implicated in fatty acid handling through epigenetically mediated transcriptional control, linking altered chromatin states to hepatic steatosis (Mondal et al., 2025). In parallel, experimental evidence suggests that metabolites such as cholestenoic acid may reduce hepatic lipid accumulation through epigenetic modulation of metabolic pathways (Wang et al., 2024). These findings support a mechanistic role for epigenetic programming in shaping metabolic phenotypes in MASLD.

Epigenetic mechanisms also intersect with other regulatory systems relevant to MASLD pathophysiology. Alterations in gut microbiota composition influence host metabolism through microbial metabolites that interact with epigenetic regulators, thereby modulating hepatic inflammation and steatosis (Takahashi et al., 2023; Mir and Ashraf, 2024; Soto et al., 2024). This bidirectional interaction highlights the integrated nature of genetic, environmental, and epigenetic factors in MASLD development.

Advances in understanding the epigenetic landscape of MASLD have important therapeutic and translational implications. Epigenetic modulation offers potential avenues for intervention, including targeting nuclear receptors such as peroxisome proliferator activated receptors to restore metabolic balance and reduce disease progression (Deshmukh et al., 2024; Li et al., 2025; Mejía-Guzmán et al., 2025). In addition, epigenetic biomarkers may enhance disease diagnosis, prognostication, and patient stratification beyond traditional clinical parameters (Abdelhameed et al., 2024).

The aim of this narrative review is to synthesize current evidence on epigenetic reprogramming in MASLD with a focus on physiological consequences and translational relevance. We examine key epigenetic mechanisms implicated in MASLD, explore how metabolic stress induces persistent epigenetic changes, discuss downstream effects on insulin resistance, inflammation, fibrosis, and disease heterogeneity, and highlight implications for reversibility, biomarker development, and precision medicine.

This schematic illustrates how epigenetic mechanisms (DNA methylation, histone modifications, and noncoding RNAs) link metabolic stress to hepatic gene expression in MASLD, contributing to disease heterogeneity and persistence. These processes affect lipid metabolism, insulin resistance, inflammation, mitochondrial dysfunction, and fibrogenesis, are influenced by lifestyle and surgical interventions, and show partial reversibility with metabolic improvement, supporting risk stratification, disease phenotyping, earlier intervention, and precision medicine.

## 2 Literature search strategy

To inform this narrative review, a targeted literature search was conducted to identify relevant studies examining epigenetic mechanisms in MASLD. Electronic databases including PubMed/MEDLINE, the Cochrane Library, and ClinicalTrials.gov were searched for publications reporting human observational studies, interventional studies, experimental animal models, and relevant systematic reviews related to epigenetic regulation in MASLD.

Search terms included combinations of keywords related to MASLD and epigenetic regulation, such as “MASLD,” “NAFLD,” “epigenetics,” “DNA methylation,” “histone modification,” “microRNA,” and “noncoding RNA.” Additional relevant studies were identified through manual review of reference lists of key articles and recent reviews.

Studies were selected based on relevance to the mechanistic and translational themes addressed in this review, with emphasis on human studies where available and experimental evidence that provides mechanistic insight into epigenetic regulation in MASLD.

## 3 Epigenetic mechanisms implicated in MASLD

**Figure 1:**
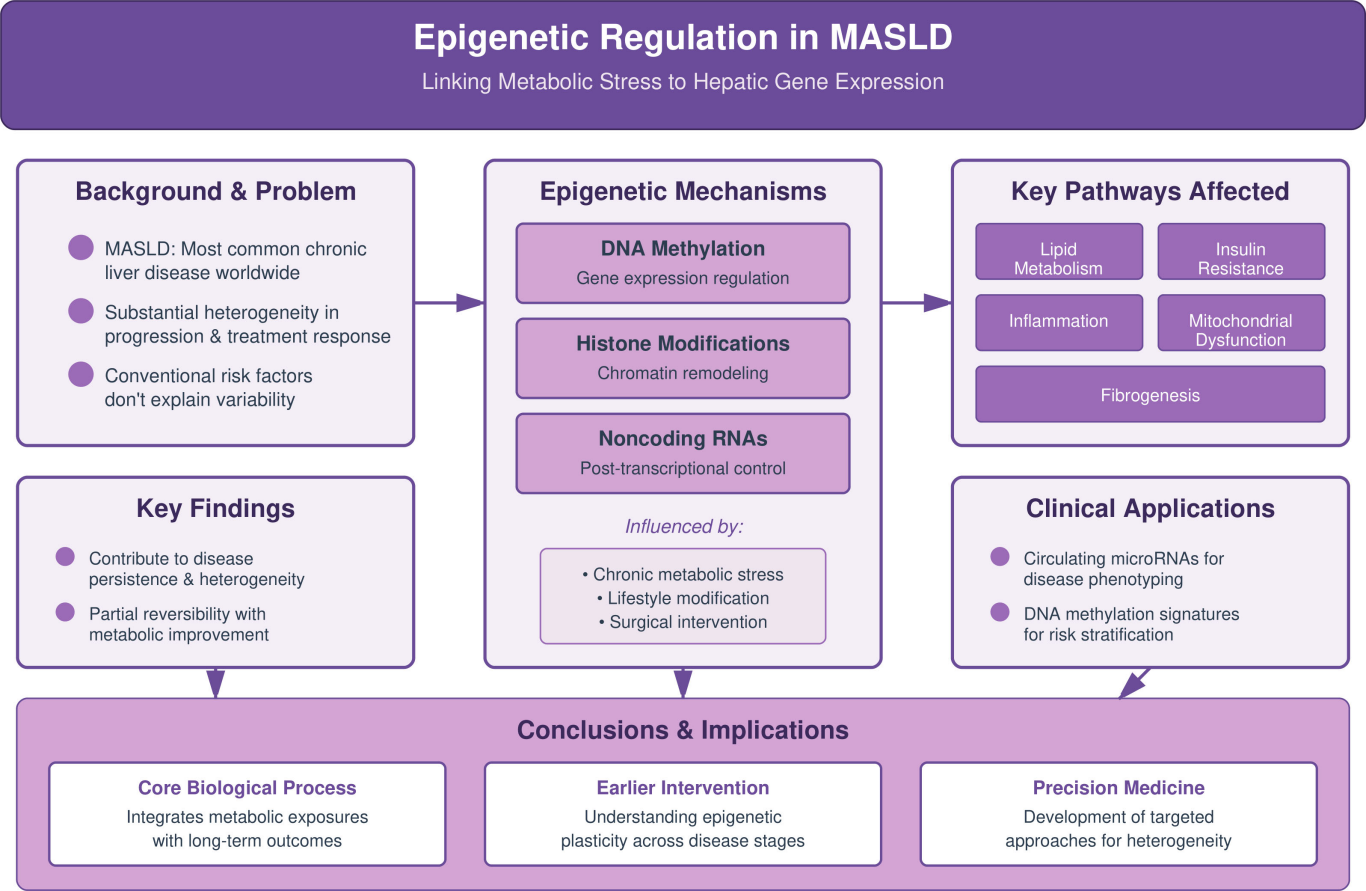
Summary diagram.

In the sections that follow, we synthesize evidence across epigenetic layers and link these mechanisms to downstream physiology and clinical translation. A schematic overview of the major epigenetic mechanisms and their physiological consequences in MASLD is presented in **Figure 1**. **Table 1** provides a translational roadmap that maps the major themes of this narrative review to potential clinical decision points in MASLD. Epigenetic regulation encompasses a set of mechanisms that alter gene expression without changing DNA sequence and it provides a central framework for understanding how metabolic and environmental cues shape hepatic biology in MASLD. These mechanisms include DNA methylation, post translational modifications of histone proteins, and regulation by noncoding RNAs such as microRNAs and long noncoding RNAs. Together they influence chromatin structure, transcriptional activity, and the stability of metabolic and inflammatory phenotypes in the liver.

**Table 1:** Translational roadmap from epigenetic reprogramming in MASLD to clinical decision making.

Review section	Core question	Epigenetic layer(s)	Key upstream drivers	Dominant physiological consequences	Clinical decision point enabled	Examples of measurable readouts
Epigenetic Mechanism(s)	What changes at the regulatory level in MASLD	DNA methylation and hydroxymethylation	Nutrient excess, insulin resistance, oxidative stress, inflammatory signaling	Stable shifts in transcription of metabolic and stress response pathways	Biomarker discovery, pathway selection	blood based methylation signatures, gene specific methylation panels
		Histone modifications, chromatin state	Metabolic intermediates, redox state, cytokine signaling	Switching between lipogenic, oxidative, and injury programs, altered cell state	Target identification, draggability mapping	chromatin enzyme activity proxies, histone related circulating signatures
		microRNAs and long noncoding RNAs	Lipotoxicity, ER stress, inflammation	Post transcriptional control of lipid metabolism, apoptosis, inflammatory tone	Noninvasive phenotyping, monitoring	circulating microRNA panels linked to steatosis activity
Metabolic stress to epigenetic reprogramming	How does metabolic dysfunction write persistent programs	Multi-layer remodeling and epigenetic memory	Visceral adiposity, insulin resistance, chronic inflammation, gut derived metabolites	Persistence of insulin resistance, inflammatory activation, stress pathway priming	Identifying high risk even when labs look improved	longitudinal shifts or stability of circulating epigenetic markers
Physiological consequences	Which pathways drive progression and why it persists	Epigenetic control of cell signaling and cell fate	Recurrent injury signals, immune crosstalk, altered nutrient sensing	Stellate activation, fibrogenesis, chronic inflammation, impaired mitochondrial function	Fibrosis enrichment, progression risk stratification	signatures associated with fibrosis stage or inflammatory activity
Heterogeneity and lean MASLD	Why patients diverge despite similar clinical features	Epigenetic integration of exposures and inherited risk	gene environment interaction, visceral fat distribution, microbiome context	Distinct phenotypes including lean disease, variable fibrosis trajectory	Phenotype assignment, tailored follow up intensity	combined genetic plus epigenetic risk layering where available
Reversibility and plasticity	What is modifiable and when	Remodeling signatures with intervention	Weight loss, exercise, dietary quality, bariatric surgery, pharmacologic therapy	Shifts in lipid handling, inflammatory tone, mitochondrial function, stress pathways	Monitoring response, defining a modifiable window	pre post intervention methylation shifts, exercise linked epigenetic changes
Precision medicine	How do we use this at bedside	Biomarker translation and therapeutic targeting	Assay feasibility, reproducibility, tissue specificity	Improved risk models, treatment selection frameworks	Screening, staging, monitoring, treatment selection	integrated models combining epigenetics with imaging and fibrosis measures

A recent narrative review by Li and colleagues synthesizes evidence that aberrant DNA methylation, histone modifications, and microRNA regulation are all involved in the development and progression of MASLD (Li et al., 2025). This work highlights how altered methylation patterns and histone marks converge on pathways that govern lipid handling, insulin signaling, oxidative stress responses, and fibrogenesis. By placing these mechanisms in a unified framework, the review supports the concept that epigenetic regulation is not a peripheral feature of MASLD but a core component of its pathobiology.

DNA methylation exerts many of its downstream effects through methyl-CpG binding proteins, which recognize methylated cytosine residues and recruit transcriptional regulatory complexes that influence chromatin structure and gene expression. Members of this family include methyl-CpG binding protein 2 (MeCP2) and proteins containing methyl-CpG binding domains (MBD1–4), which act as functional interpreters of DNA methylation signals. Experimental studies have shown that these proteins participate in regulating hepatic stellate cell activation, inflammatory signaling, and fibrogenic pathways—processes central to the pathogenesis of MASLD and its progressive form, metabolic dysfunction–associated steatohepatitis (MASH). For example, MeCP2 has been implicated in the transcriptional regulation of genes involved in stellate cell activation and extracellular matrix remodeling during liver fibrosis, highlighting how methylation-dependent chromatin regulation may translate metabolic stress signals into sustained fibrogenic responses. These observations reinforce the concept that DNA methylation–associated regulatory proteins act as key intermediaries linking epigenetic marks to downstream cellular phenotypes relevant to disease progression.

Histone based mechanisms have received particular attention. Min and coauthors summarize post translational modifications of histone proteins that regulate lipid metabolism, inflammation, oxidative stress, and cell death in MASLD (Min et al., 2025). They describe how changes in histone methylation, acetylation, phosphorylation, and ubiquitination can alter chromatin accessibility at promoters and enhancers of metabolic genes, thereby shifting hepatocyte transcriptional programs toward steatosis, inflammatory activation, or fibrotic remodeling. These observations suggest that histone modifications act as important sensors and mediators of metabolic stress in the liver.

At a more granular level, specific histone acetylation programs have been linked to transcriptional activation of key lipogenic genes in MASLD models. In experimental MASLD settings, lipogenic transcriptional activation has been associated with promoter-proximal enrichment of acetylation marks, including increased H3K9 acetylation at genes involved in lipid synthesis and metabolic remodeling (Morral et al., 2021). In a mechanistic study focused on fatty acid synthase, histone modification patterns at FASN were shown to shift in association with carbohydrate-responsive element binding protein–linked regulation, supporting a direct chromatin-level mechanism coupling nutrient sensing to lipogenesis (Cai et al., 2018). Complementing these promoter-level observations, enhancer activation programs have also been implicated: high-fat-diet MASLD models demonstrate dysregulated gene expression linked to H3K27 acetylation at regulatory regions (Ma et al., 2022) hibition of histone acetyltransferase activity, consistent with chromatin opening that reinforces lipogenic transcriptional networks. Interventional evidence further supports functional relevance, as pharmacologic inhibition of histone acetyltransferase activity attenuated MASLD phenotypes *in vivo* and *in vitro* (Chung et al., 2019), consistent with histone acetylation as a modifiable driver of lipogenic reprogramming. Together, these findings provide mechanistic specificity to the broader concept that histone modifications act as metabolic sensors that can stabilize lipogenic gene expression programs under sustained nutrient excess.

Noncoding RNAs provide an additional layer of epigenetic control that is directly relevant to MASLD. In a case control study of patients with nonalcoholic fatty liver disease, Sookoian and colleagues identified a variant in a long noncoding RNA that was associated with more severe disease and with adverse cardiometabolic traits (Sookoian et al., 2017). This finding supports the view that long noncoding RNAs participate in the regulation of metabolic and inflammatory pathways that influence both hepatic and systemic phenotypes. In parallel, Hendy and coauthors evaluated circulating microRNAs as noninvasive biomarkers of MASLD and reported that serum levels of microRNA 122 and microRNA 34a were increased in patients with disease and correlated with histologic severity (Hendy et al., 2019). Although primarily designed as a biomarker study, the work reinforces the idea that microRNA networks are altered in fatty liver and reflect underlying changes in hepatic transcriptional control.

Taken together, these studies illustrate the main epigenetic modalities that operate in MASLD. DNA methylation and histone modifications shape chromatin states and transcriptional potential, while noncoding RNAs fine tune gene expression and link hepatic changes to systemic circulation. This mechanistic foundation provides the context for understanding how chronic metabolic stress can drive persistent epigenetic reprogramming in MASLD, which is the focus of the next section.

## 4 Metabolic stress leading to epigenetic reprogramming in MASLD

**Figure 2:**
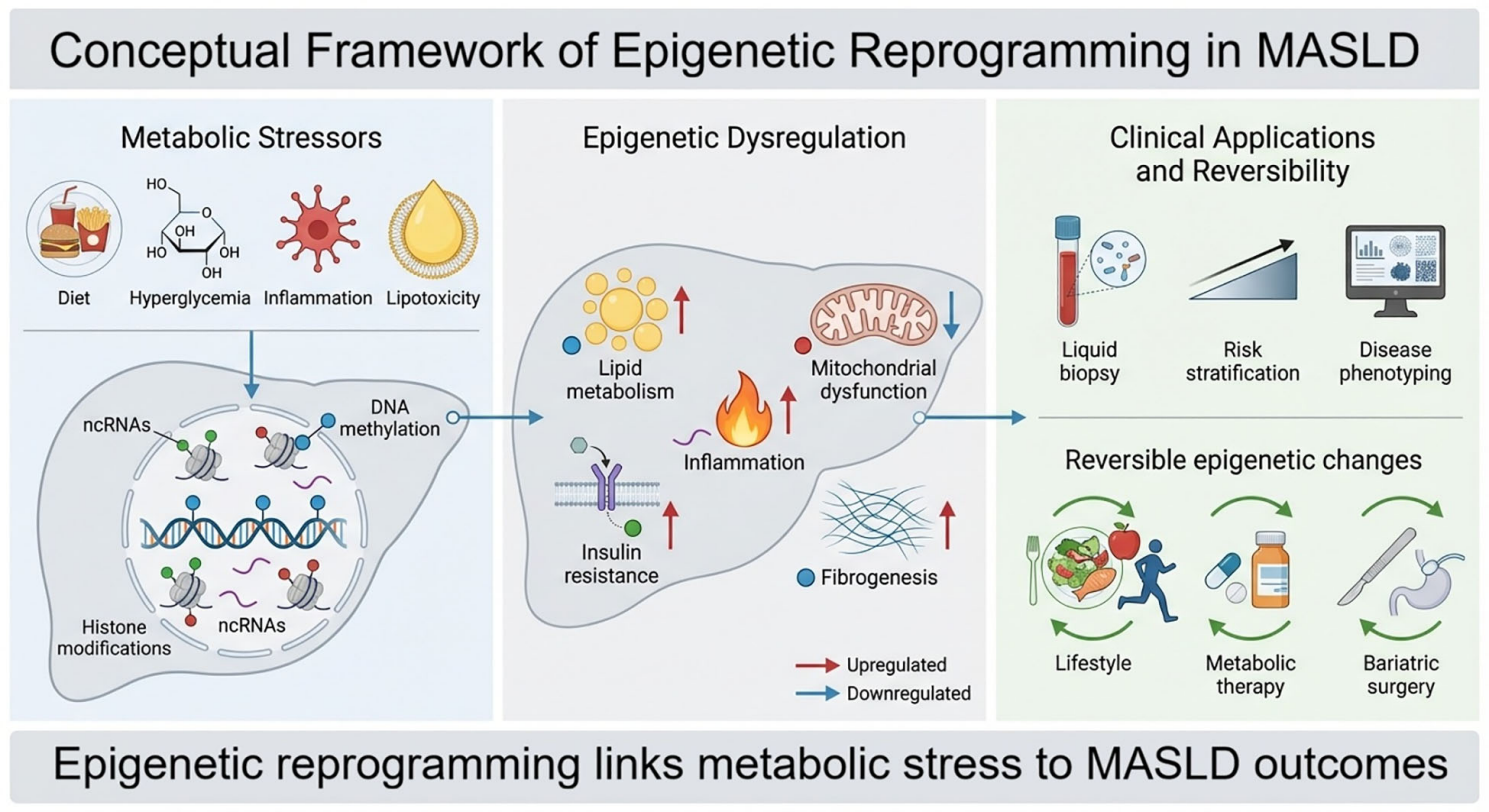
Metabolic stress–driven epigenetic reprogramming in MASLD. Metabolic stressors such as diet, hyperglycemia, inflammation, and lipotoxicity alter DNA methylation, histone modifications, and noncoding RNA regulation, affecting lipid metabolism, insulin resistance, inflammation, mitochondrial dysfunction, and fibrogenesis.

Chronic metabolic stress is a defining feature of MASLD and represents a major driver of epigenetic reprogramming in hepatic and extrahepatic tissues (**Figure 2**). Obesity, insulin resistance, dyslipidemia, mitochondrial dysfunction, and gut derived metabolic signals exert sustained pressure on cellular regulatory systems, leading to durable changes in DNA methylation, chromatin organization, and non-coding RNA expression. Importantly, some of these epigenetic alterations may persist beyond the initial metabolic insult, creating a form of “metabolic memory” in which prior exposure to nutrient excess or inflammatory stress leaves a durable regulatory imprint on hepatic gene expression. This concept suggests that even when metabolic parameters improve, epigenetically stabilized transcriptional programs may continue to influence disease trajectory.

Evidence from human observational studies supports the association between metabolic stress and altered DNA methylation in MASLD. In an observational study, Mwinyi and colleagues demonstrated that MASLD related metabolic stress is associated with significant DNA methylation changes near transcription start sites of genes involved in lipid transport, cholesterol metabolism, energy balance, and vitamin D pathways (Mwinyi et al., 2017). These methylation shifts were linked to altered lipoprotein composition and vitamin D metabolism and were more pronounced in the presence of fibrosis, suggesting that sustained metabolic stress induces epigenetic remodeling that may contribute to disease progression.

More direct evidence of active epigenetic remodeling in response to metabolic stress was provided by Barchetta and colleagues in a cross-sectional observational study of patients with MASLD (Barchetta et al., 2025). The authors showed that global markers of active DNA demethylation in peripheral blood mononuclear cells were increased in individuals with MASLD and were associated with higher fibrosis risk. Gene specific hypomethylation of SOCS3 and altered methylation and expression of SREBF1 and TXNIP were linked to inflammatory signaling, oxidative stress, and fibrogenic pathways. These findings indicate that metabolic stress in MASLD is associated not only with static methylation changes but also with active and ongoing epigenetic reprogramming.

Non coding RNAs appear to act as sensitive responders to metabolic stress in early disease stages. In a population based clinical study, He and colleagues reported that circulating microRNA 29b levels were elevated in patients with MASLD and correlated with intrahepatic lipid content, triglycerides, and fasting glucose independently of age, sex, body mass index, and diabetes status (He et al., 2019). This suggests that microRNA alterations may reflect metabolic stress before overt liver injury becomes apparent. Similarly, in a cross-sectional study of biopsy proven MASLD, Salvoza and colleagues showed that serum microRNA 34a and microRNA 122 were significantly elevated and correlated with dyslipidemia, particularly very low-density lipoprotein cholesterol and triglycerides (Salvoza et al., 2016). These findings support a role for microRNAs as epigenetic mediators linking lipid overload to hepatic stress responses.

Several narrative reviews provide a broader mechanistic framework for how metabolic stress induces epigenetic reprogramming in MASLD. Ha and colleagues emphasized that metabolic stress and gut derived metabolites act as epigenetic modifiers that reshape host gene expression through DNA methylation, histone modifications, and non-coding RNA regulation, thereby influencing lipid metabolism, inflammation, fibrosis, and hepatocarcinogenesis (Ha et al., 2025). Similarly, Aiello and colleagues described a bidirectional relationship in which metabolic reprogramming alters epigenetic enzymes and chromatin states, while epigenetic changes reinforce lipogenesis, inflammation, insulin resistance, and ferroptosis, particularly in the context of MASLD associated hepatocellular carcinoma (Aiello et al., 2025).

Additional narrative reviews highlight specific metabolic stress pathways that converge on epigenetic regulation. Caputo and colleagues focused on mitochondrial dysfunction as a central driver of MASLD and described how metabolic stress affects mitochondrial DNA methylation and non-coding RNA circuits that regulate oxidative stress and energy metabolism (Caputo et al., 2023). Miramon and colleagues further emphasized that diet, lifestyle, and metabolic imbalance shape epigenetic marks that influence disease severity and progression, reinforcing the concept that epigenetic reprogramming reflects cumulative metabolic exposures rather than isolated insults (Irigaray-Miramon, 2025). Exercise related metabolic stress has also been shown to induce beneficial epigenetic remodeling. In a narrative review, Zhang and colleagues synthesized evidence that physical activity triggers changes in DNA methylation, histone modifications, and non-coding RNA expression that improve hepatic lipid metabolism, reduce inflammation, and enhance mitochondrial function, providing a mechanistic basis for sustained benefits of lifestyle interventions (Zhang et al., 2025).

A defining feature of metabolic memory is the relative stability of certain epigenetic marks despite partial metabolic normalization (Chen et al., 2024). In MASLD, emerging data suggests that not all disease associated methylation or chromatin changes fully revert with short term improvement in glycemic control or weight reduction. For example, disease linked methylation signatures have been shown to correlate strongly with fibrosis stage, raising the possibility that as fibrosis advances, epigenetic programs become progressively stabilized (Gerhard et al., 2018; Johnson et al., 2021). While interventional studies such as those following bariatric surgery demonstrate significant remodeling of the hepatic methylome, complete normalization to control patterns is not consistently observed, indicating selective plasticity rather than full erasure (Ahrens et al., 2013).

This partial reversibility suggests that epigenetic regulation in MASLD operates along a spectrum from highly dynamic marks responsive to metabolic fluctuation to more stable modifications that encode prior metabolic exposure. Such stability may help explain clinical observations in which fibrosis risk or inflammatory activity persists despite improvements in body weight or insulin sensitivity. However, longitudinal data directly demonstrating durable epigenetic imprinting in MASLD remain limited, and distinguishing true metabolic memory from slow biological reversal represents an important area for future investigation.

## 5 Physiological consequences of epigenetic alterations in MASLD

Epigenetic reprogramming in MASLD has important physiological consequences that influence disease persistence, progression, and severity. By stabilizing transcriptional programs related to metabolism, inflammation, and tissue remodeling, epigenetic alterations translate metabolic stress into sustained functional changes within the liver.

Human observational studies provide evidence that epigenetic changes are linked to clinically relevant inflammatory and fibrotic pathways. Barchetta and colleagues demonstrated that markers of active DNA demethylation in peripheral blood mononuclear cells were increased in individuals with MASLD and were associated with higher fibrosis risk (Barchetta et al., 2025). In this study, altered methylation of genes such as SOCS3, SREBF1, and TXNIP correlated with inflammatory signaling, oxidative stress, and fibrogenic activity, suggesting that epigenetic regulation contributes directly to pathological immune and metabolic responses rather than simply reflecting disease severity.

Epigenetic dysregulation also appears to influence hepatic lipid handling and insulin sensitivity, key physiological features of MASLD. Studies examining DNA methylation and non-coding RNA profiles have linked altered epigenetic states to disrupted lipid metabolism, mitochondrial dysfunction, and impaired energy homeostasis, which together promote hepatic steatosis and metabolic inflexibility (Mwinyi et al., 2017; Caputo et al., 2023). These changes may help explain why insulin resistance and lipid accumulation persist even in the absence of progressive weight gain.

MicroRNA mediated regulation further contributes to downstream physiological effects. Circulating microRNAs associated with lipid metabolism and cellular stress responses have been shown to correlate with intrahepatic fat content and metabolic parameters in MASLD. Several microRNAs have been repeatedly implicated, including miR-122 and miR-34a, which are associated with hepatic lipid accumulation, inflammation, and disease severity, as well as miR-29b, which correlates with intrahepatic lipid content, triglyceride levels, and glucose metabolism (Salvoza et al., 2016; He et al., 2019). Although often studied as biomarkers, these microRNAs are also functionally involved in regulating inflammatory and fibrogenic pathways within the liver.

Collectively, these findings support the concept that epigenetic alterations in MASLD have tangible physiological consequences that extend beyond gene regulation. By reinforcing insulin resistance, chronic inflammation, and fibrogenic activation, epigenetic reprogramming contributes to disease persistence and progression. This physiological imprint provides a mechanistic basis for clinical heterogeneity in MASLD and sets the stage for understanding distinct disease phenotypes, including lean MASLD, which are discussed in the following section.

## 6 MASLD heterogeneity and lean MASLD

MASLD is a biologically heterogeneous condition, with substantial variability in steatosis burden, inflammatory activity, fibrosis risk, and long-term outcomes that is not fully explained by body mass index or conventional metabolic markers. MASLD is defined by hepatic steatosis in the absence of significant alcohol exposure, and it spans a spectrum from simple steatosis to NASH with progressive fibrosis and downstream complications (Niriella et al., 2019; Buzova et al., 2020). Although MASLD is strongly linked to obesity and type 2 diabetes, it also occurs in individuals with normal body mass index, supporting the concept that multiple biological routes can converge on a similar hepatic phenotype (Petersen, 2010; Wei et al., 2015; Feldman et al., 2017; Fracanzani et al., 2017; Hagström et al., 2018).

Our group’s prior systematic review synthesized available evidence on the genetic and epigenetic determinants of lean MASLD and highlighted wide variability in prevalence estimates, in part reflecting inconsistent definitions of “lean” across studies and populations (Shen et al., 2014; Lin et al., 2022; Stasinou et al., 2022). Lean disease has been conceptually categorized into a more common subtype associated with visceral adiposity and insulin resistance, and another linked to monogenic steatosis, reinforcing that lean MASLD is not a single entity (Chatterjee et al., 2021; Chahal et al., 2022). This framework helps reconcile why some lean patients resemble classic metabolic MASLD while others appear to develop steatosis through distinct inherited or molecular drivers.

Across populations, the most consistent signal supporting heterogeneity in lean disease is genetic susceptibility, particularly PNPLA3. In a population study using proton magnetic resonance spectroscopy and transient elastography, non-obese MASLD patients had a higher frequency of the PNPLA3 rs738409 risk allele compared with obese MASLD, and the variant remained an independent factor associated with MASLD in non-obese individuals (Wei et al., 2015). In a community cohort, the PNPLA3 rs738409 risk allele accounted for fatty liver in individuals without metabolic syndrome and remained associated after adjustment for dietary pattern and metabolic factors, with stronger effects among those without metabolic syndrome (Shen et al., 2014). Similar associations have been observed across diverse settings including pediatric cohorts and multiple adult populations (Honda et al., 2016; Feldman et al., 2017; Fracanzani et al., 2017; Niriella et al., 2019; Stasinou et al., 2022). Some studies also suggest that the effect size of PNPLA3 may be amplified in lean phenotypes. For example, in lean individuals, PNPLA3 rs738409 GG genotype was more prevalent among MASLD cases than in heavier categories, and the genotype was associated with the highest relative risk in lean strata (Lin et al., 2022).

TM6SF2 also contributes to heterogeneity, with evidence that its effects may vary by body weight and lipid phenotype. Exome wide association and candidate variant studies have implicated TM6SF2 in MASLD susceptibility and histologic severity in general MASLD populations (Kozlitina et al., 2014; Sookoian et al., 2015). In lean focused analyses, TM6SF2 rs58542926 has been linked to lean specific lipid patterns, including lower serum triglycerides in lean carriers, which may partially explain why some lean MASLD patients present with steatosis despite less overt dyslipidemia (Lin et al., 2022). Large scale analyses such as the UK Biobank based study further reinforce that lean MASLD is associated with visceral adipose tissue and metabolic markers as well as genetic variants including PNPLA3 rs738409 and TM6SF2 rs58542926, supporting multifactorial risk architecture (Chahal et al., 2022).

Importantly, heterogeneity in lean MASLD is clinically meaningful because outcomes are not uniformly benign. In a long term follow up cohort of biopsy proven MASLD, lean patients did not have increased overall mortality compared with overweight patients, but they had a significantly higher risk of developing severe liver disease over follow up (Hagström et al., 2018). In another biopsy proven cohort, lean MASLD patients had lower prevalence of hypertension, diabetes, metabolic syndrome, NASH, and stage F2 or higher fibrosis compared with overweight or obese MASLD, yet visceral obesity remained an important modifier of hepatic and cardiovascular injury (Fracanzani et al., 2017). These findings collectively support the notion that lean MASLD encompasses both lower risk and higher risk subgroups, likely reflecting different combinations of adipose distribution, metabolic context, and inherited susceptibility.

In contrast to the relatively mature genetic literature, direct evidence for epigenetic determinants specific to lean disease remains sparse. In our systematic review, Buzova and colleagues represented the only included study directly interrogating an epigenetic signal in lean disease, reporting depletion of circulating histone variants macroH2A1.1 and macroH2A1.2 in lean MASLD (Buzova et al., 2020). While preliminary, this finding supports biological plausibility for chromatin level mechanisms contributing to lean phenotypes and highlights a major knowledge gap that future multi omics studies should address.

Beyond PNPLA3 and TM6SF2, less frequent genetic associations further support etiologic heterogeneity. CETP polymorphisms were associated with fatty liver risk in adolescent females independent of adiposity, suggesting that lipid transfer pathways may predispose to steatosis even in lean individuals (Adams et al., 2012). APOC3 gain of function variants were associated with marked hypertriglyceridemia, impaired triglyceride clearance, insulin resistance, and a high prevalence of MASLD in lean Asian Indian men (Petersen, 2010). Finally, genome wide association work in lean MASLD suggested an HLA locus signal and further proposed an interaction between host genotype and gut microbiome features, offering an additional pathway by which lean disease biology may diverge across individuals and populations (Yoshida et al., 2020).

## 7 Reversibility and plasticity of epigenetic changes

A defining feature of epigenetic regulation is its plasticity. Unlike fixed DNA sequence variation, epigenetic marks can shift in response to changes in metabolic state, environmental exposures, and therapeutic interventions. This creates an important clinical premise in MASLD: if epigenetic reprogramming helps sustain steatosis, inflammation, or fibrogenic pathways, then interventions that improve metabolic health may also recalibrate disease relevant epigenetic programs and potentially modify long term risk.

Evidence from interventional settings suggests that at least a portion of the MASLD epigenome is modifiable. Ahrens and colleagues reported distinct DNA methylation signatures in MASLD and showed remodeling patterns after bariatric surgery, supporting the concept that major weight loss and metabolic improvement can be accompanied by measurable shifts in methylation and gene regulation rather than purely transient biochemical change (Ahrens et al., 2013). This aligns with the broader idea that interventions may not only reduce hepatic fat content but may also change upstream regulatory states that influence disease persistence.

Lifestyle interventions, particularly exercise, may provide a mechanistic route to epigenetic remodeling. In a narrative review, Zhang and colleagues summarized evidence that physical activity induces changes in DNA methylation, histone modifications, and non-coding RNA regulation that align with improved hepatic lipid metabolism, reduced inflammatory signaling, enhanced mitochondrial function, and improved autophagy, offering a plausible biological basis for sustained benefits beyond short term changes in weight or glycemia (Zhang et al., 2025). This is particularly relevant for MASLD given the frequent clinical observation that cardiometabolic improvements can occur even with modest weight change.

Dietary composition may also modulate epigenetic states, including through availability of methyl donors and bioactive compounds that interact with epigenetic enzymes and mitochondrial function. Murphy and colleagues described how diets enriched in methyl donors can alter epigenetic landscapes, supporting the concept that nutritional exposures can influence DNA methylation patterns relevant to metabolic disease biology (Murphy et al., 2013). Complementing this, Caputo and colleagues highlighted that bioactive compounds such as resveratrol and related polyphenols may influence mitochondrial associated epigenetic regulation and oxidative stress pathways, suggesting one potential route by which dietary patterns could contribute to epigenetic normalization in MASLD (Caputo et al., 2023). While the strength of clinical evidence varies across specific compounds, these data collectively support diet as a modulator of epigenetic programming rather than solely a caloric exposure.

Epigenetic marks in MASLD are not fixed. The interventional literature, from bariatric surgery to lifestyle change, suggests that at least some of the regulatory changes tied to steatosis and inflammation can move in a healthier direction when the metabolic environment improves (Ahrens et al., 2013; Zhang et al., 2025). The open question is not whether change is possible, but when it is most achievable and which signals track meaningful clinical benefit rather than short lived fluctuation. That uncertainty is exactly why epigenetic readouts are attractive clinically: if they can be measured reliably, they may help identify who is still in a modifiable window and who needs earlier, more intensive therapy (Murphy et al., 2013; Caputo et al., 2023).

## 8 Precision medicine implications

Epigenetic biology in MASLD becomes clinically useful when it helps answer practical questions that routine clinical variables do not resolve, such as who is likely to progress, who has occult fibrotic risk despite modest metabolic features, and who might benefit from earlier or more intensive intervention. Because epigenetic marks integrate environmental exposures and metabolic history, they are well positioned to support risk stratification and patient phenotyping, particularly when MASLD heterogeneity limits the predictive value of body mass index, aminotransferases, or single time point metabolic measures. The potential clinical applications of epigenetic information in MASLD are summarized in **Table 2**, emphasizing use case driven opportunities rather than validated tools. Representative epigenetic regulators implicated in MASLD pathogenesis, including key proteins and noncoding RNAs, are summarized in **Table 3**.

**Table 2:** Clinical use cases of epigenetic information in MASLD.

Clinical question	Epigenetic signal type	Sample source	Potential clinical utility	Key limitations
Who is at highest risk for disease progression	DNA methylation signatures	Peripheral blood	Early risk stratification beyond conventional metabolic markers	Interindividual variability and lack of standardized thresholds
How active is inflammatory liver injury	Dynamic epigenetic modifications	Blood or liver tissue	Noninvasive assessment of disease activity and inflammatory burden	Temporal variability and limited validation
Will a patient respond to lifestyle or pharmacologic intervention	Reversible epigenetic changes	Blood	Monitoring biological response to intervention before histologic change	Confounding by concurrent metabolic exposures
What is the likelihood of fibrosis progression	Stable epigenetic patterns	Blood or liver tissue	Prognostic enrichment for patients at risk of advanced fibrosis	Limited longitudinal data
Can treatment durability be assessed over time	Persistence or reemergence of epigenetic marks	Blood	Identification of sustained versus transient therapeutic effects	Sensitivity to environmental re exposure

**Table 3:** Key epigenetic regulators implicated in MASLD pathogenesis.

Epigenetic regulator	Epigenetic mechanism	Key biological pathways affected	Relevance to MASLD/MASH
DNMT1, DNMT3A, DNMT3B	DNA methylation of CpG sites	Lipid metabolism, inflammatory signaling	Altered hepatic gene expression and metabolic dysregulation
MeCP2, MBD proteins	Binding to methylated DNA and recruitment of chromatin remodeling complexes	Hepatic stellate cell activation, extracellular matrix regulation	Promotion of fibrogenesis and fibrosis progression
Histone acetyltransferases (HATs)	Histone acetylation and chromatin opening	Lipogenic gene transcription	Increased hepatic lipid accumulation
Histone deacetylases (HDACs)	Histone deacetylation and chromatin condensation	Inflammatory and metabolic gene regulation	Modulation of metabolic stress responses
miR-122	microRNA-mediated post-transcriptional regulation	Lipid metabolism, hepatocyte injury	Associated with steatosis severity and metabolic dysregulation
miR-34a	microRNA regulation of stress and apoptotic pathways	Inflammation, apoptosis	Linked to disease severity and metabolic stress
miR-29b	microRNA regulation of metabolic signaling	Lipid metabolism and glucose regulation	Correlates with intrahepatic lipid content and triglycerides

A large body of work has focused on circulating microRNAs as noninvasive biomarkers that reflect hepatic injury and metabolic stress. In a cross-sectional study of biopsy proven MASLD, Salvoza and colleagues reported that serum miR 34a and miR 122 were elevated in MASLD and correlated with VLDL cholesterol and triglycerides, supporting potential utility for identifying disease and tracking metabolic injury without biopsy (Salvoza et al., 2016). Complementing this, Yamada and colleagues found that several circulating microRNAs, including miR 122 and miR 34a, were elevated in MASLD and that miR 122 correlated with steatosis severity, reinforcing feasibility for disease monitoring (Yamada et al., 2013). In a population based clinical study, He and colleagues reported that serum miR 29b correlated with intrahepatic lipid content and triglycerides, suggesting potential value for detecting early steatosis and metabolic stress signals before advanced injury is apparent (He et al., 2019). For severity phenotyping, Muangpaisarn and colleagues reported that serum miR 34a correlated with MASLD activity score and fibrosis, with higher levels in more active disease, supporting a role in distinguishing inflammatory phenotypes relevant to escalation decisions (Muangpaisarn, 2016). Taken together, these studies support a layered use case where microRNAs can contribute to screening, staging enrichment, and longitudinal monitoring alongside imaging and laboratory markers.

Beyond diagnosis, epigenetic biomarkers may also support prognostication. In a retrospective cohort of Japanese MASLD patients, Akuta and colleagues reported that serum miR 122 levels were associated with disease severity and that integrating miR 122 with clinical features and fibrosis staging improved mortality risk stratification, illustrating how an epigenetic readout might add prognostic signal beyond standard measures (Akuta et al., 2020). At a population screening level, Ando and colleagues reported that lower circulating miR 20a and miR 27a were associated with severe MASLD and appeared to reflect intrahepatic fat accumulation rather than fibrosis, suggesting potential utility for identifying high burden steatosis phenotypes in broader populations (Ando et al., 2019).

DNA methylation signatures also provide a plausible bridge from mechanism to clinical translation. Murphy and colleagues reported widespread methylation changes across the MASLD spectrum, with differential methylation correlating with altered gene expression in pathways related to fibrosis, inflammation, wound healing, and one carbon metabolism, supporting the premise that methylation profiles could inform stage discrimination and progression biology (Murphy et al., 2013). More recently, Barchetta and colleagues reported that active demethylation marks in peripheral blood mononuclear cells correlated with MASLD presence and fibrosis risk, with gene specific patterns linked to inflammatory signaling and metabolic stress pathways, strengthening the case for blood based epigenetic markers as scalable tools for risk enrichment (Barchetta et al., 2025). System level syntheses further consolidate these signals. A meta-analysis by Liu and colleagues concluded that miR 122 helps distinguish MASLD from controls and that miR 34a can differentiate NASH from simple steatosis, while also noting variability in serum to liver concordance that must be addressed before clinical deployment (Liu et al., 2018). A systematic review by Zhang and colleagues similarly highlighted recurrent associations for specific methylation loci and microRNAs across studies, supporting biomarker plausibility while underscoring the need for harmonized assays and validated thresholds (Zhang et al., 2021).

Precision medicine also includes treatment stratification and target selection. Reviews focused on MASLD related hepatocellular carcinoma emphasize that epigenetic regulators are not only biomarkers but also potential drug targets that map onto clinically meaningful pathways such as fibrogenesis, inflammation, ferroptosis, and tumor aggressiveness (Aiello et al., 2025). In parallel, Carpi and colleagues reviewed microRNA directed therapeutics including mimics and anti microRNA strategies and summarized early translational programs aimed at attenuating fibrosis and tumorigenic progression, illustrating a plausible future where epigenetic targeting supports individualized risk reduction strategies in high risk MASLD phenotypes (Carpi et al., 2024).

The main takeaway is that epigenetic information is already closest to clinic in two areas: noninvasive phenotyping and risk enrichment using circulating microRNAs and methylation based signatures, and longer-term therapeutic stratification as epigenetic targets mature. The immediate need is not more candidate markers, but robust validation across diverse populations, alignment with clinically actionable outcomes, and integration into multi parameter models that combine epigenetics with imaging, fibrosis measures, and metabolic context (Liu et al., 2018; Akuta et al., 2020; Zhang et al., 2021).

A critical challenge for clinical translation is the degree to which peripheral epigenetic signatures reflect the intrahepatic landscape. Epigenetic regulation is inherently tissue specific, and DNA methylation patterns or noncoding RNA profiles measured in peripheral blood mononuclear cells or serum may capture systemic inflammatory or metabolic signals rather than hepatocyte-specific transcriptional states (Zhou et al., 2017). While several studies demonstrate associations between circulating microRNAs and histologic severity, concordance between blood-based epigenetic markers and intrahepatic epigenomic remodeling remains variable and incompletely characterized (Liu et al., 2018). Direct paired analyses comparing liver tissue and peripheral samples are limited, and differences in cell composition, signal dilution, and dynamic turnover may further complicate interpretation (Husby, 2020). Addressing this tissue specificity gap through integrated liver–blood profiling studies will be essential before peripheral epigenetic markers can be confidently deployed for precision risk stratification or therapeutic monitoring in MASLD.
